# Mesenchymal Stem Cells Enhance Pulmonary Antimicrobial Immunity and Prevent Following Bacterial Infection

**DOI:** 10.1155/2020/3169469

**Published:** 2020-03-28

**Authors:** Wenchao Li, Weiwei Chen, Saisai Huang, Xiaojun Tang, Genhong Yao, Lingyun Sun

**Affiliations:** Nanjing Drum Tower Hospital, The Affiliated Hospital of Nanjing University Medical School, Nanjing, Jiangsu 210008, China

## Abstract

**Background:**

Immunosuppressants such as cyclophosphamide (CTX) have been employed to treat a wide array of autoimmune diseases. The most unfavourable side effects of these drugs are their suppression on the antimicrobial immunity and increasing the risk of infection. As a promising substitution/adjunct, mesenchymal stem cells (MSCs) are currently being tested in several clinical trials. However, their influence on the recipients' antimicrobial immunity remains unclear.

**Methods:**

In this study, C57BL/6 mice were treated with either CTX or MSCs, and then both the innate and adaptive immunity of the lung were determined. To investigate the influence of CTX and MSCs on the immune defence against infection, the treated mice were intranasally infected with opportunistic pathogen *Haemophilus influenzae* (Hi). Bacterial clearance and antibacterial immune responses were analysed.

**Results:**

Our data showed that CTX strongly inhibited the proliferation of lung immune cells, including alveolar macrophages (AMs) and T cells, whereas MSCs increased the numbers of these cells. CTX suppressed the phagocytic activity of AMs; on the contrary, MSCs enhanced it. Notably, infusion of MSCs led to a remarkable increase of regulatory T cells and Th1 cells in the lung. When infected by Hi, CTX did not significantly impair the elimination of invaded bacteria. However, MSC-treated mice exhibited accelerated bacterial clearance and moderate inflammation and tissue damage.

**Conclusion:**

Our study reported that unlike traditional immunosuppressants, modulation of MSCs on the recipient's immune response is more elegant. It could preserve and even enhance the antimicrobial defence, suggesting that MSCs are better choice for patients with high risk of infection or those who need long-term immunosuppressive regimen.

## 1. Background

Cyclophosphamide (CTX) is one of the commonly used immunosuppressants and has been employed to treat a variety of autoimmune disorders, including but not limited to, systemic lupus erythematosus (SLE), systemic sclerosis, some forms of vasculitis, rheumatoid arthritis, and severe aplastic anemia [1]. As a nitrogen mustard prodrug, CTX undergoes hepatic conversion to form active metabolites which subsequently cause cell death by leading to inter- and intrastrand DNA crosslinking. Rapidly proliferating cells are more susceptible to CTX [2]. Consequently, the normal immune cells are also disproportionately affected, which in turn might increase the patient's susceptibility to infection [3, 4]. Indeed, it has been reported widely by clinical studies that severe infection is the leading cause of death in patients with autoimmune diseases, which was partly due to the long-term usage of immunosuppressive drugs [5–7]. Thus, new drugs or therapy which is less deleterious to patients' normal immune responses is in urgent need.

Mesenchymal stem cells (MSCs) are a type of nonhematopoietic, adult stem-like cell that can be isolated from various tissues [8]. In addition to their tissue repairing potential, MSCs obtain potent immunosuppressive capacities. Therefore, they have been largely investigated and tested as a novel therapeutic tool for several clinical applications, including different rheumatic diseases [9]. In response to inflammatory mediators released from activated immune cells (i.e., IFN-*γ* and TNF-*α*), MSCs regulate the function of a broad range of immune cells [9]. The mechanisms involved in their immunomodulatory activity are still under exploration but rely on both cell-cell contact and paracrine effects through secreting soluble factors including hepatocyte growth factor, prostaglandin-E2 (PGE2), TGF-*β*, HLA-G5, or indoleamine 2,3-dioxygenase (IDO) [10]. Except for the direct effect of these soluble factors, MSCs may also regulate immune responses by educating immune cells to a regulatory phenotype, including regulatory T cells (Tregs) or anti-inflammatory macrophages (M2) [11, 12]. Continuous usage of immunosuppressive drugs will dampen the anti-infection immune responses of patients; however, for MSCs, little is known. Notably, in a long-term retrospective study, Liang et al. reported that 7.7% of patients received MSCs exhibited a remarkably decreased incidence and/or duration of respiratory tract infection after infusion of MSCs compared with that before infusion [13], suggesting that MSCs may enhance the recipients' antimicrobial immunity. Thus, it is important to access the safety of MSCs to see whether they are the better choice for the patients with high susceptibility to infection.

Considering that most of the MSCs entered the lung and are trapped after intravenous injection [14, 15], in this study, we chose the pulmonary infection model to compare the direct influences of CTX and MSCs on the recipient's anti-infection immunity. *Haemophilus influenzae* (Hi) is a Gram-negative coccobacillus, which colonizes asymptomatically in the upper respiratory tract of healthy people. When host immunity is dysregulated, it can disseminate into privileged anatomical locations and cause a wide spectrum of diseases including pneumonia [16]. Moreover, Hi are frequently isolated from patients who received immunosuppressive drugs. Thus, we chose this opportunistic bacterium to assess the antimicrobial immunity of the recipients.

## 2. Methods

### 2.1. Animals

Age-matched female C57BL/6 mice (6~8-week-old; Nanjing Medical University Animal Core) were used in all experiments. Animals were housed in the animal facility of Nanjing Drum Tower Hospital.

### 2.2. Ethics Approval

The animal protocol was approved by the Ethics Committee for Animal Research in the Affiliated Drum Tower Hospital. All animal procedures were approved by the Ethics Committee for Animal Research in the Affiliated Drum Tower Hospital under the approved protocol code No. 20160802. This study has been conducted under the guideline of the Guide for the Care and Use of Laboratory Animals.

### 2.3. Preparations of MSCs

MSCs were isolated as described in our previous study [17]. Briefly, umbilical cords from scheduled healthy caesarean sections were collected and were cut into 1 cm long pieces and finely minced and digested with CDH buffer (250 U/ml collagenase II (Sigma), 100 U/ml dispase, and 100 U/ml hyaluronidase in DMEM/F12 medium) at 37°C on an orbital shaker for 4 h. The suspension was then diluted at 1 : 5 with PBS at room temperature and centrifuged at 840 g for 10 min. Cells were plated at 4000–6000 cells per cubic centimetre in MSC media, and nonadherent cells and debris were removed after 48 h. Adherent cells were cultured, and passage 6-8 cells were used in all experiments. Phenotype of the resulting MSCs was determined by surface staining ([Supplementary-material supplementary-material-1]) [18].

### 2.4. Treatment with CTX and MSCs

For CTX treatment, mice were injected with CTX (40 mg/kg, i.p.) twice, at a five-day interval. For MSC infusion, each mouse received 1 × 10^6^ cells once via intravenous route. The detailed regimen is shown in the figures.

### 2.5. Preparation of Bacteria

*H. influenzae*, strain 86-028NP, was grown on brain heart infusion agar plate supplemented with nicotinamide adenine dinucleotide (5 *μ*g/ml) and hemin (10 *μ*g/ml) (sBHI) at 37°C in the CO_2_ incubator overnight. Then, the colonies were inoculated into sBHI broth and subjected to shaking to reach OD600 = 0.45. Bacteria were then harvested, washed, and resuspended in PBS at a concentration of 3 × 10^9^ CFU/ml.

### 2.6. Pulmonary *H. influenzae* Infection Model

Mice were anaesthetized by intraperitoneal injection of 100 *μ*l ketamine/xylazine (150 mg/10 mg/kg) and inoculated with 1 × 10^8^ CFU of prepared bacteria in 50 *μ*l PBS intranasally. To enumerate bacteria in the lung, mice were sacrificed 24 hours later. Bronchial alveolar lavage fluid (BALF) and the lungs were collected. The left lobes of the lungs were homogenized in 1 ml sterile PBS. Tenfold serial dilutions of the homogenates and BALF were prepared in PBS and plated on sBHI plates. Colonies were counted after overnight culture at 37°C.

### 2.7. Isolation of BALF and Lung Cells

The trachea was cannulated with a 20-gauge catheter. The lungs were then flushed with 1.0 ml cold PBS twice; total returns averaged 1.4-1.8 ml. BALF was centrifuged at 400 g for 5 min. Supernatants were aliquoted and stored at -80°C. The cell pellet was resuspended in 500 *μ*l PBS. Twenty microliter of the cell suspension was processed for cell counts with the automated cell counter (Shanghai Ruiyu Biotech Co., Ltd.). To isolate lung cells, the lungs were minced and incubated in digestion buffer (Isocove's DMEM containing 3.5 mg/ml collagenase A (Roche) and 2.5 mg/ml DNase I (Sigma)) for 1 hour at 37°C and then mashed through a 40 *μ*m cell strainer (BD Falcon). Then the fragments were pressed through the strainer with the plunger end of a 5 ml syringe. The red cells were removed by lysing solution (BD Pharmingen).

### 2.8. BALF Cytokine Measurements

Cytokines in the BALF were determined by ELISA kits (Biolegend or R&D Systems) for TNF-*α*, IL-6, MCP-1, and KC. The detecting limitation of MCP-1 is 30 pg/ml, while that of TNF-*α*, IL-6, and KC is 7.5 pg/ml.

### 2.9. Lung Morphometric Analysis

The lungs were fixed in 10% formalin. The paraffin-embedded lungs were then cut into 5 *μ*m thick sections and subsequently stained with hematoxylin and eosin (H&E) for histological analysis. Five sections from the same lobes of each sample were randomly chosen, and the mean linear intercept (MLI) was then caculated as previously described [19]. Briefly, an image of each examined section was digitally captured at ×40 magnification. Ten 10 cm horizontal lines at 2 cm intervals were then used to count alveolar surface intersections. The MLI was calculated by the following equation: the sum of the length of all counting lines divided by the total number of counted intercepts of alveolar septa.

### 2.10. Phagocytic Activity Determination

The phagocytic activity of alveolar macrophages was determined by the Vybrant™ Phagocytosis Assay Kit (Thermo Fisher). According to the manufacturer's instruction, BALF cells were collected from the lungs and cultured in the 96-plat bottom-well plates in the presence of fluorescein-labelled *Escherichia coli* K-12 BioParticles. One hour later, the BioParticle loading suspension from all of the microplate wells was removed by vacuum aspiration. The extracellular fluorescence probe was then quenched off by trypan blue. The microplate was read by the fluorescence plate reader (Spark® Multimode Microplate Reader, Tecan).

### 2.11. Intracellular Cytokine Staining and Flow Cytometry

BALF cells were stained at 4°C in PBS containing 1% FBS after Fc*γ*RII/III blockade with anti-mouse CD16/CD32 (clone 93; eBioscience). Surface staining was performed with antibodies purchased from eBioscience (anti-CD45, clone 30-F11; anti-CD11b, clone M1/70; anti-CD11c, clone N418; anti-Ly6G (Gr-1), clone RB6-8C5; anti-CD3, clone 145-2C11; anti-CD4, clone RM4-5; and anti-CD44, clone IM7). For Treg staining, cells were surface-stained with anti-CD4 and anti-CD25 (clone PC61.5) and followed by permeabilization with the Foxp3/Transcription Factor Buffer Staining Set (eBiosciences). Then the cells were stained with anti-foxp3 (clone FJK16s) antibody. For intracellular staining, cells were surface-stained with anti-CD4 and anti-CD44, followed by permeabilization with the Fixation/Permeabilization Solution Kit (BD). Then the cells were stained with anti-IFN-*γ* (Clone XMG1.2), anti-IL-17 (Clone eBio17B7), and anti-IL-4 (Clone 11B11). The apoptotic status of lung cells was detected with the Apoptosis Detection kit (BD Pharmingen) following the instructions of the manufacturer. In this method, apoptosis was examined by the Annexin V/7-AAD double staining. All samples were analysed with FACSCalibur and FACSFortessa. Data were analysed with FlowJo.

### 2.12. Statistical Analysis

All analyses were performed with GraphPad. Data were analysed by unpaired *t* test. The Kruskal-Wallis test was used to evaluate variance among all groups. If a significant variance was found, the Mann-Whitney test was used to determine significant differences between individual groups. *p* < 0.05 was considered to represent statistically significant difference.

## 3. Results

### 3.1. CTX Reduced Cells in the Lung, whereas MSCs Increased Them

**Figure 1:**
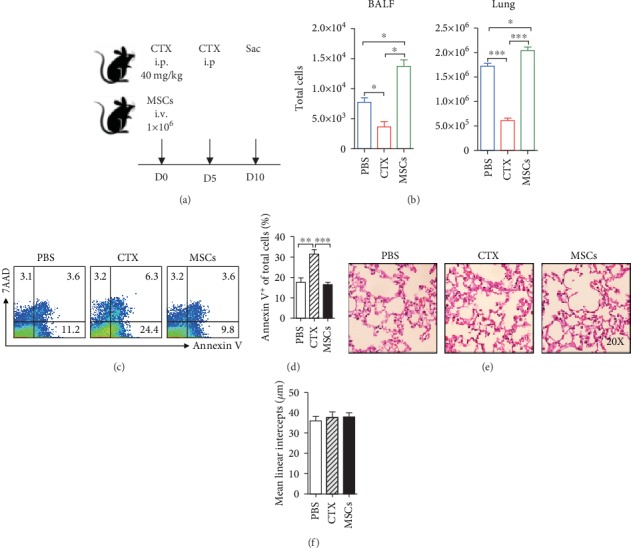
CTX reduced cells in the lung, whereas MSCs increased them. (a) B6 mice were treated with MSCs or CTX and sacrificed at indicated time. i.p.: intraperitoneal; i.v.: intravenous. (b) Cells from the BALF and lungs were isolated and counted. (c, d) Apoptotic status of lung cells was determined by FACS. (e) Lung pathology was examined by H&E staining. (f) Quantitative analyses of the pulmonary alveolar sizes, as measured by MLI. Data were expressed as the means ± SEM. *n* = 3 mice for each treatment group; ^∗^*p* < 0.05; ^∗∗^*p* < 0.01; ^∗∗∗^*p* < 0.001. This experiment is representative of three individual experiments.

To compare the direct influences of CTX and MSCs on the lung immunity, we treated mice with CTX or MSCs as depicted in Figure 1(a). The dose of CTX was equal to that used for pulse treatment for SLE patients, and that of MSCs was the same as that reported previously [20]. PBS-treated mice were set as controls. Since CTX is toxic to the proliferating cells, we first compared treatment-induced changes of the total lung cell number. More than one-half reductions of cells were observed in both the BALF and lungs of CTX-treated mice. However, a reverse phenomenon was observed in the mice receiving MSCs. After being injected with MSCs, lung cells increased dramatically, especially in the alveolar space (BALF) (Figure 1(b)). Next, we confirmed that the reduction of cells was caused by increased cell death, as CTX-treated mice had 2-fold more apoptotic cells (Annexin V^+^) in the lung (Figures 1(c) and 1(d)). Most of the cells which underwent apoptosis were T cells and macrophages ([Supplementary-material supplementary-material-1]). The observation of increased cell death made us determine whether CTX will exhibit deleterious effects on the normal structure of the lungs. As H&E staining shown, no obvious abnormality was detected in either of the treated groups (Figure 1(e)). The changes in peripheral pulmonary alveolar size were also quantified by morphometric measurement of mean linear intercept (MLI). No significant difference of MLI was detected between groups (Figure 1(f)).

### 3.2. MSCs Increased Both Quantity and Phagocytic Activity of Alveolar Macrophages

**Figure 2:**
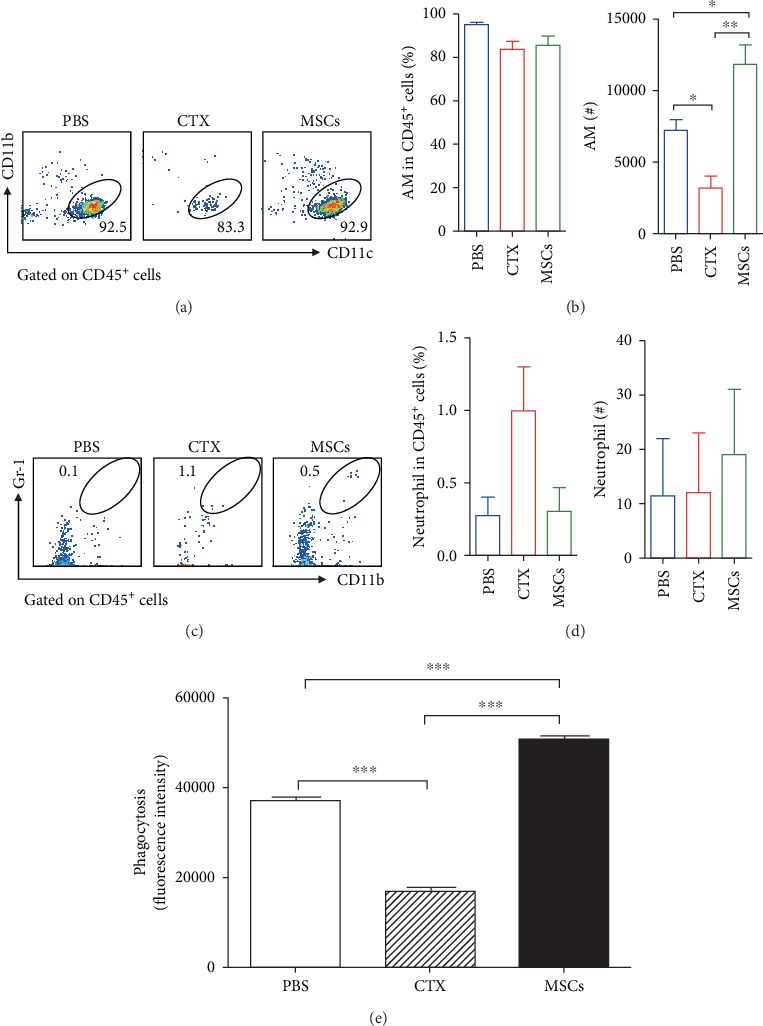
The number and phagocytic ability of alveolar macrophages were increased in the MSC-treated mice. Mice were treated with MSCs or CTX as described above. Cells in the BALF were collected and analysed by surface staining. The frequencies and absolute numbers of alveolar macrophages (CD11b^−^CD11c^+^) (a, b) and neutrophils (CD11b^+^Gr-1^+^) (c, d) were determined by surface staining. (e) The phagocytic ability of AMs was determined. Data were expressed as the means ± SEM. *n* = 3 mice for each treatment group. ^∗^*p* < 0.05; ^∗∗^*p* < 0.01. This experiment is representative of three individual experiments.

Next, we analysed the immune cells of the lungs to identify the cell subset affected most by the treatments. FACS data showed that in all three groups, more than 90% of the BALF cells were alveolar macrophages (AMs) (Figure 2(a)); nearly no neutrophils were detected (Figures 2(c) and 2(d)). However, when looked at the absolute numbers of AMs, a remarkable decrease was detected in the mice that received CTX, whereas MSCs increased AMs (Figure 2(b)). AMs play an important role in eliminating the invading pathogens by direct phagocytosis. Thus, we next examined whether the phagocytic activity of AMs was affected by CTX or MSCs. The data showed that CTX strongly inhibited the phagocytosis of bacteria by AMs whereas MSCs enhanced it (Figure 2(e)), implying that infusion of MSCs may strengthen the recipient's defence against infection.

### 3.3. Treatment with MSCs Significantly Increased Tregs in the Lung

**Figure 3:**
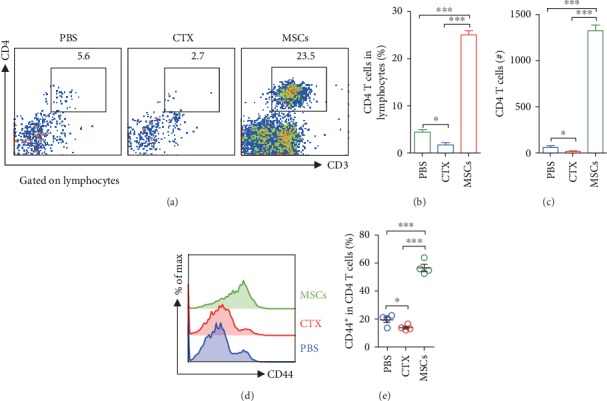
Treatment with MSCs significantly increased CD4 T cells in the lung. Mice were treated with MSCs or CTX as described above. BALF cells were collected and analysed by surface staining. (a) Representative FACS data of BALF CD4 T cells. The frequencies (b) and absolute numbers (c) of CD4 T cells. (d, e) Expression of CD44 by CD4 T cells. Data were expressed as the means ± SEM. *n* = 3-4 mice for each treatment group; ^∗^*p* < 0.05 and ^∗∗∗^*p* < 0.001. This experiment is representative of three individual experiments.

**Figure 4:**
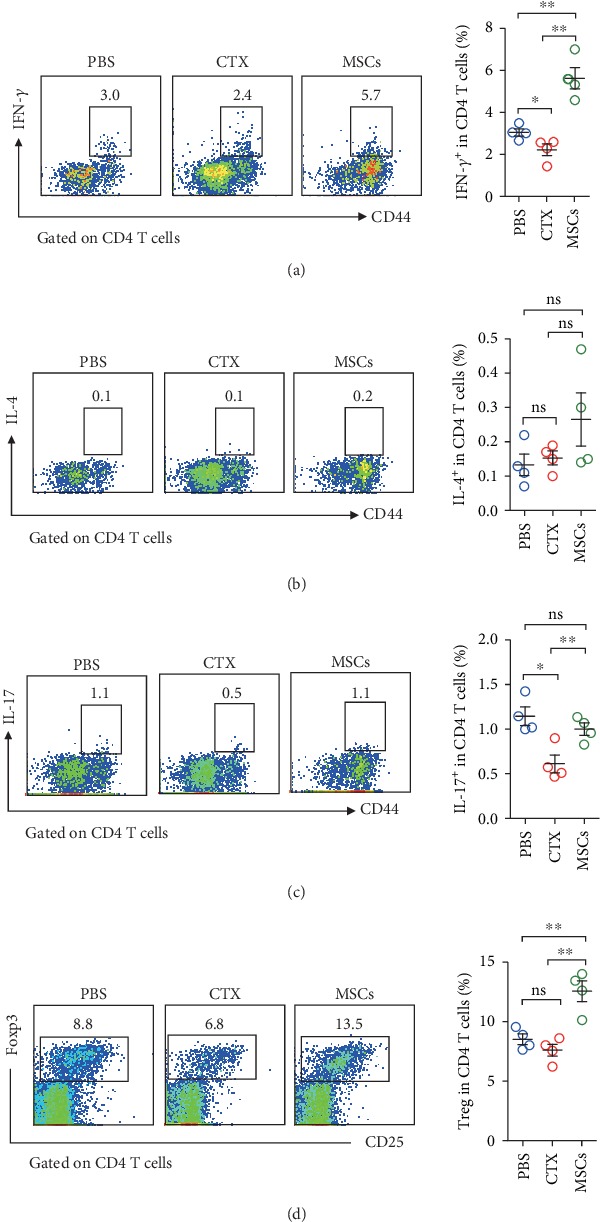
Treatment with MSCs significantly increased Treg and Th1 cells in the lung. Mice were treated with MSCs or CTX as described above. Lung cells were isolated and stimulated with PMA and ionomycin for five hours. Cytokine production by CD4 T cells was determined by intracellular staining. Percentages of IFN-*γ* (a), IL-4 (b), and IL-17 (c) producing cells were shown. (d) Tregs in the lung were determined by intracellular staining. Data were expressed as the means ± SEM. *n* = 4 mice for each treatment group. ^∗^*p* < 0.05; ^∗∗^*p* < 0.01; ns: no significant difference. This experiment is representative of three individual experiments.

In addition to innate cells, T cells are also required for the defence against infection. We observed that MSCs significantly increased CD4 T cells in the lung, whereas CTX inhibited the proliferation of CD4 T cells (Figures 3(a)–3(c)). Next, we compared the functions of the CD4 T cells from different groups. FACS data showed that the majority of CD4 T cells in the lungs of MSC-treated mice had an activated CD44^hi^ phenotype. For those treated with CTX, only a small group of CD4 T cells expressed CD44, which was comparable with the control mice (Figures 3(d) and 3(e)). According to the previous studies, based on the cytokine production, CD4 T cells can be generally subgrouped into Th1 (IFN-*γ*), Th2 (IL-4), and Th17 (IL-17) cells. Thus, we next examined the influences of MSCs and CTX on the functions of CD4 T cells. We found that most of the CD4 T cells from all the three groups produced IFN-*γ*, exhibiting Th1 phenotype. Infusion of MSCs significantly increased the percentage of Th1 cells, whereas CTX inhibited it (Figure 4(a) and [Supplementary-material supplementary-material-1]). Few Th2 cells were detected in all the mice. Neither MSCs nor CTX affected Th2 cells (Figure 4(b)). For Th17 cells, MSCs had no influences on them, but CTX exhibited suppressive effects on them (Figure 4(c)). It has been widely reported that MSCs exert their immunosuppressive functions by inducing regulatory T cells (Tregs). In line with these studies, a remarkable increase of Tregs was only observed in the MSC-treated lungs (Figure 4(d)). Consistent with FACS data, we detected increases of IFN-*γ* and TGF-*β*, which were produced by Th1 and Tregs in the BALF of MSC-treated mice ([Supplementary-material supplementary-material-1]).

### 3.4. MSCs Provided Protection against Following Pulmonary Bacterial Infection

**Figure 5:**
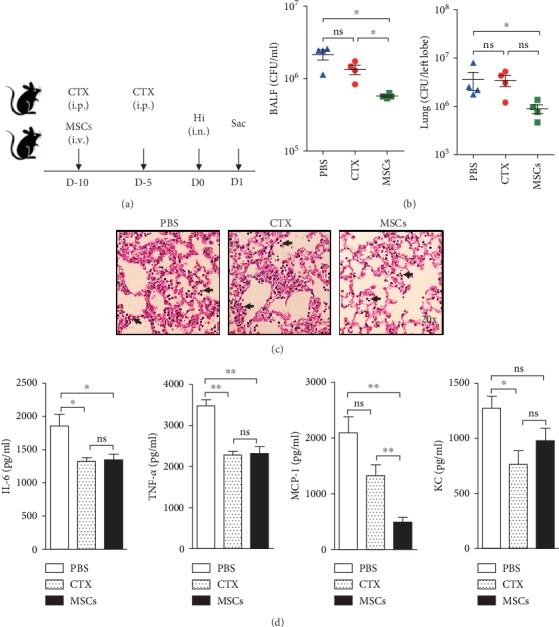
Pretreatment with MSCs provides protection against pulmonary bacterial infection. (a) MSC- or CTX-treated mice were infected by 1 × 10^8^ CFU of Hi. PBS-treated mice were used as controls. (b) Twenty-four hours later, mice were sacrificed and bacteria in the BALF and lung were numerated. (c) Lung pathology was examined by H&E staining. Arrowheads indicated infiltrated neutrophils. (d) Inflammatory-related cytokines IL-6 and TNF-*α* and chemokines MCP-1 and KC in the BALF collected from infected mice were measured by ELISA. Data were expressed as the means ± SEM. *n* = 4 mice for each treatment group; ^∗^*p* < 0.05; ^∗∗^*p* < 0.01; ns: no significant difference. This experiment is representative of three individual experiments.

Next, we assessed the capability of the modified lung immunity to fight against infection. All the mice were infected with Hi intranasally and sacrificed 24 hours later (Figure 5(a)). Then, the bacterial clearance and inflammatory responses were determined. Our data showed that MSCs strongly inhibited the growth of Hi in the lung, but CTX did not (Figure 5(b)). According to the H&E staining data, infection induced a dramatic increase of lung cells in control and CTX-treated mice; however, MSC-treated mice had fewer infiltrating cells (Figure 5(c)). Besides, a thickened alveolar wall was only observed in the CTX and PBS groups. Then, we determined chemokines and proinflammatory cytokines which are responsible for the recruitment of immune cells to the lung. We found that productions of inflammatory cytokines TNF-*α* and IL-6 were significantly inhibited by CTX and MSCs (Figure 5(d)). Monocyte chemotactic protein 1 (MCP-1) plays a role in the recruitment of monocytes to sites of injury and infection. Here, we showed that both CTX and MSCs could downregulate its secretion by lung cells, but MSCs exhibited stronger inhibition, which may explain the reduced cell infiltration. KC is a major neutrophil chemoattractant. Only CTX showed a slightly suppressive effect on it. Thus, both CTX and MSCs can suppress the infection-induced production of inflammatory cytokines. These data showed that unlike CTX, MSCs could reduce the severity of infection.

### 3.5. Mice Treated with MSCs Had Fewer Neutrophils but More Alveolar Macrophages in the Lung after Infection

**Figure 6:**
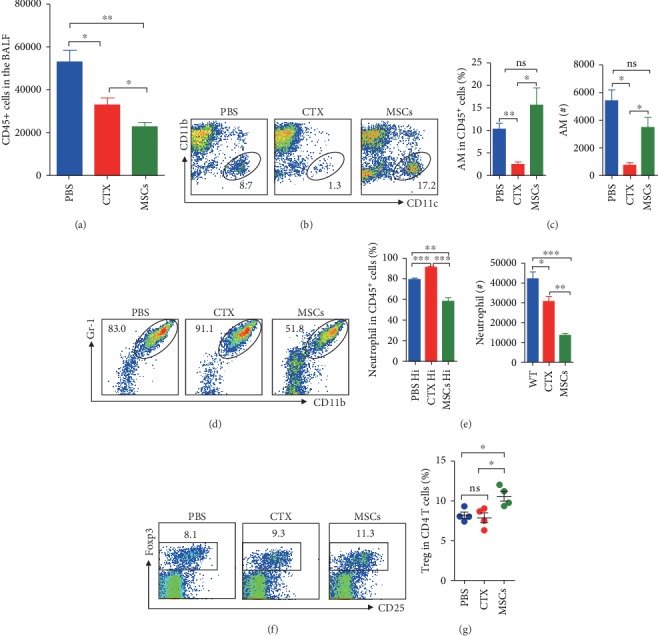
Mice treated with MSCs had fewer neutrophils but more alveolar macrophages in the lung after infection. Mice treated with MSCs or CTX were infected with Hi and sacrificed as described above. BALF cells were collected for analyses. (a) The number of BALF CD45^+^ cells (leukocytes). (b, c) Alveolar macrophages (CD11b^−^CD11c^+^) and (d, e) neutrophils (CD11b^+^Gr-1^+^) were determined by surface staining, and both their percentages of leukocytes and absolute numbers were shown. (f, g) Tregs in the lungs were determined by intracellular staining. Data were expressed as the means ± SEM. *n* = 4 mice for each treatment group; ^∗^*p* < 0.05; ^∗∗^*p* < 0.01; ^∗∗∗^*p* < 0.001; ns: no significant difference. This experiment is representative of three individual experiments.

Consistent with the H&E staining data, after infection by Hi, increases of leukocytes in the lung were observed in all the three groups (compared with the uninfected mice, Figure 1(a)). In the BALF, the numbers of leukocytes were significantly lower in both the CTX- and MSC-treated groups, whereas the most dramatic decrease was detected in the MSC-treated group (Figure 6(a)). After infection, the percentages of AMs dropped down dramatically in all the three groups. A large amount of neutrophils infiltrated into the lungs and replaced the AMs, becoming the majority of lung cells, especially in the control and CTX-treated mice (~80% and 95%, respectively) (Figures 6(b) and 6(c)). The same as that was seen in the uninfected mice, CTX mice had significantly lower AMs. However, in the MSC-treated mice, both the frequencies and absolute numbers of neutrophils were lower (Figures 6(d) and 6(e)), indicating that MSCs could suppress the recruitment of neutrophils and possibly prevent tissue damages caused by overactivated inflammatory responses. When looking at Tregs, we found that MSC-treated mice still had a higher percentage of these cells, which may be responsible for the suppressed inflammation (Figures 6(f) and 6(g)). For other Th cells, no significant differences were observed ([Supplementary-material supplementary-material-1]).

## 4. Discussion

Immunosuppressive drugs such as cyclophosphamide, methotrexate, and glucocorticoids have exerted tremendous impact on ameliorating autoimmune diseases [1]. However, these agents are associated with adverse off-target effects, which can result in an alarming increase of infection rates. Due to their profound immunosuppressive capability, mesenchymal stem cells (MSCs) are currently being considered a novel adjunct to conventional therapy or a standalone therapy to treat the autoimmune diseases [8]. Nevertheless, little is known about their influence on the antimicrobial immunity. In this study, employing the pulmonary bacterial infection model, we examined and compared the effects of the high-dose CTX and MSC treatments on the murine antibacterial immunity. We found that high-dose CTX dramatically inhibited the proliferation of immune cells and suppressed their functions, including phagocytic ability and cytokine production, but it did not compromise the recipient's ability to kill the invading bacteria. Interestingly, for MSCs, our data showed that they could strengthen the immune defence against infection. We observed that MSCs dramatically enhanced the phagocytosis of bacteria by alveolar macrophages. Moreover, MSC treatment induced an increase of Tregs in the lung and suppressed the infection-induced inflammation, which may reduce the tissue damage. Thus, our data show that unlike traditional cytostatic immunosuppressive drugs, MSCs do not exert any detrimental effects on the normal immune system; on the contrary, it could provide protection.

Innate cells are the first line of immune defence against the invading pathogens. In the lung, by direct phagocytosis and killing, alveolar macrophages (AMs) are the main force that fights against infection during the early stage. In an elegant study by Santosuosso et al., they showed that after receiving a dose of 150 mg CTX/kg of body weight, the number of AMs reduced gradually and began to restore two weeks after treatment [21]. CTX dramatically inhibited the proliferation of AMs but did not affect their production of TNF-*α* and NO, which are required for bacterial killing. In our study, although we used a more moderate treatment, we still observed a profound reduction of AMs. Moreover, phagocytosis of bacteria by AMs was impaired by CTX. These data were consistent with the *in vitro* observation by Yang et al. They assessed the cytotoxicity of CTX with RAW264.7 macrophage cells [22]. A large amount of studies have reported that CTX is equally distributed through the blood after injection and depletes lymphocytes. It reduces both B and T lymphocytes, but preferentially affects CD4 T cells [2]. For the local immune responses, less is known. In our study, we observed that intraperitoneally injected CTX also reduced the number of CD4 T cells in the lung (Figure 3). According to the immunologic data gathered from multiple sclerosis studies, CTX may drive the immune system away from a T helper type 1 (Th1) immune that is deleterious in MS towards a more favourable T helper type 2 (Th2) profile [23, 24]. Moreover, cytokines associated with the Th2 response are increased in CTX-treated patients [24]. CTX has also been shown to encourage a Th2 phenotype and reverse increased IFN-*γ* production of CD8 T cells in patients with secondary progressive MS [25]. However, in this study, where we used the healthy mice to access the safety of CTX, the resulting data are different. We found that CTX did not affect either the Th2 response or Tregs in the lung (Figure 4). It exhibited inhibitory effects on Th1 and Th17 responses, indicating that it might weaken the mucosal immunity. Our data showed intraperitoneal injection of CTX dramatically suppressed both the innate and adaptive immune responses, but it did not significantly impair the clearance of opportunistic *H. influenzae*. This could be due to the recruited neutrophils, which migrate rapidly from the circulation to the lung and carry out bacterial phagocytosis and killing, as after infection, the number of neutrophils was only slightly reduced by CTX.

After intravenous injection, the accumulation of MSCs in the lungs has been widely reported. Their short survival time and limited distribution to other sites suggest that MSC rapidly passes on their effect to resident cells, which may subsequently mediate the immunomodulatory and regenerative effect induced by MSC administration [14, 26]. Here, we found that MSC treatment induced an increase of alveolar macrophages and T cells in the lung. It has been shown in several disease models that MSCs could polarize macrophages to the immunosuppressive phenotype (M2) via secretome [27–29]. However, in line with Mei et al.'s study in treating sepsis with MSCs, we showed that when transferred to normal mice, MSCs enhanced the phagocytic ability of AMs (Figure 2(e)), suggesting that MSCs could promote the antimicrobial immunity [30]. The divergent effects of MSCs on the macrophages could be explained by the difference of environment. It has been described that MSCs can be polarized *in vitro* towards either anti-inflammatory or proinflammatory phenotypes, depending on the TLR ligand time/concentration used for activation [31, 32].

Induction of Tregs is a pivotal mechanism by MSCs to exert immunosuppressive functions. Mechanisms involved in Treg induction by MSCs have been dug deeply [33, 34]. The study by Akiyama et al. showed that intravenous transfer of mouse bone marrow MSCs to naive mice induced T cell apoptosis in peripheral blood and bone marrow. The apoptotic T cells then triggered macrophages in the spleen to produce TGF-*β* that subsequently results in the upregulation of Tregs in peripheral blood [35]. Braza et al. raised that after phagocytosis of MSCs, monocyte displayed immunosuppressive phenotype and can secrete IL-10 or TGF-*β* to induce differentiation of Tregs [36]. In this study, we indeed observed a dramatic increase of the lung Tregs after MSC infusion (Figure 4). Previous studies suggest that Tregs could keep the delicate balance of allowing for effective antipathogenic immune responses and preventing immune pathology during acute infection. However, whether the preexisting Treg in the lung is a friend or a foe remains unknown. Here, our data show that MSC treatment significantly inhibit the production of inflammatory cytokines and recruitment, and this could be attributed to the modulation by Tregs. But it could also be a result of quick clearance of bacteria by macrophages, which prevent the trigger of severe inflammation.

## 5. Conclusions

In conclusion, our data showed that unlike traditional immunosuppressant, the modulation of MSCs on the recipient's immune response is more elegant. It could preserve and even enhance the host's antimicrobial defence by augmenting phagocytic ability of macrophages. Moreover, by induction of Tregs, MSCs also help to prevent the overactivation of the infection-induced inflammation. Thus, our data suggest that MSCs are better choice for patients with high risk of infection.

## Data Availability

The datasets generated and/or analysed during the current study are included within the article and are available from the corresponding authors on reasonable request.

## Supplementary Materials

Supplementary Materials Figure S1: phenotype of isolated umbilical cord MSCs. MSCs were isolated as described in Methods. Expression of MSC markers was determined by flow cytometry. Figure S2: CTX-induced apoptosis of lung leukocytes. Figure S3: treatment with MSCs significantly increased Treg and Th1 cells in the BALF. Figure S4: MSC increased production of IFN-*γ* and TGF-*β*. Figure S5: lung Th responses of infected mice.
