# Supplementary material for: Mesenchymal Stem Cells Enhance Pulmonary Antimicrobial Immunity and Prevent Following Bacterial Infection
Source: Stem Cells Int. 2020 Mar 28;2020:3169469. doi: 10.1155/2020/3169469 (PMC7142356; doi:10.1155/2020/3169469)
Supplement: Supplementary Materials — Figure S1: phenotype of isolated umbilical cord MSCs. MSCs were isolated as described in Methods. Expression of MSC markers was determined by flow cytometry. Figure S2: CTX-induced apoptosis of lung leukocytes. Figure S3: treatment with MSCs significantly increased Treg and Th1 cells in the BALF. Figure S4: MSC increased production of IFN-γ and TGF-β. Figure S5: lung Th responses of infected mice. [file 3169469.f1.docx]

**
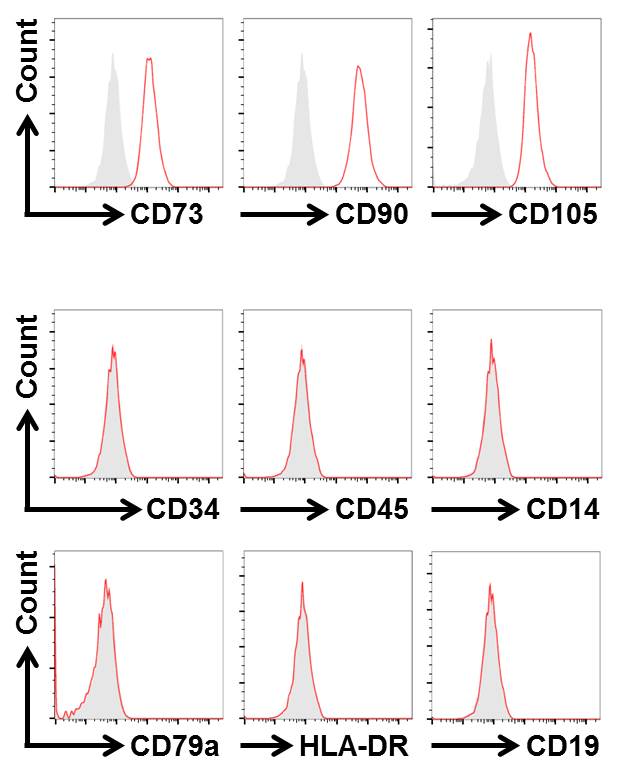
**

**Figure S1. Phenotype of isolated umbilical cord MSCs.** MSCs were isolated as described in the method. Expression of MSC markers were determined by flow cytometry.

**
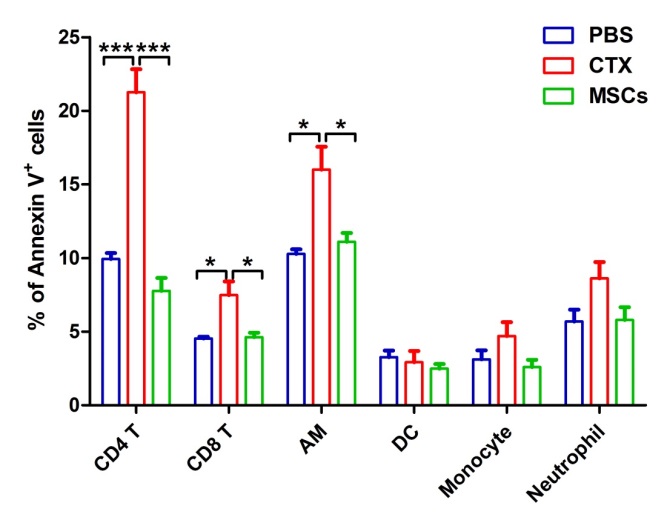
**

**Figure S2. CTX induced apoptosis of lung leukocytes.** B6 mice were treated with MSCs or CTX and sacrificed at indicated time. i.p., intraperitoneal; i.v., intravenous. Cells from lungs were isolated. Apoptotic status of lung leukocytes was determined by FACS. Data were expressed as means ± SEM. n=4 mice for each treatment group; *, *p*<0.05; ***, *p*<0.001.

**
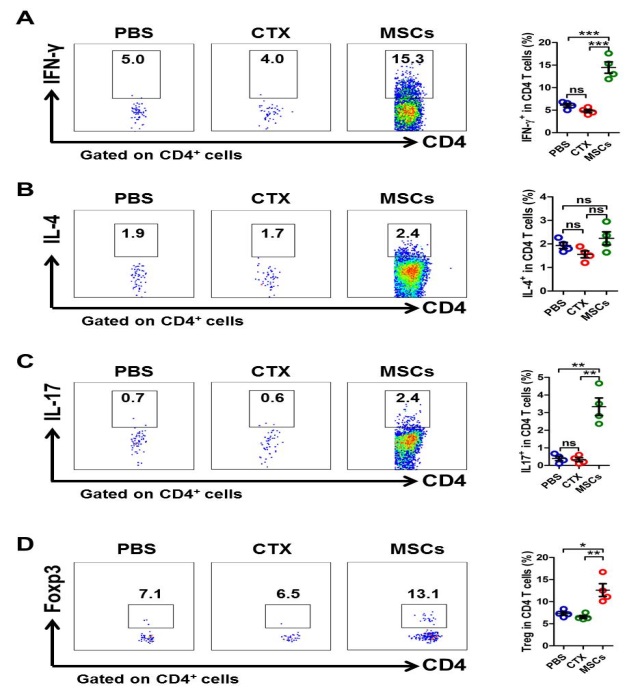
**

**Figure S3. Treatment with MSCs significantly increased Treg and Th1 cells in the BALF.** Mice were treated with MSCs or CTX as described above. BALF cells were isolated and stimulated with PMA and ionomysin for five hours. Cytokine production by CD4 T cells was determined by intracellular staining. Percentages of IFN-γ (A), IL-4 (B) and IL-17 (C) producing cells were shown. (D) Tregs were determined by intracellular staining. Data were expressed as means ± SEM. n=4 mice for each treatment group; *, *p*<0.05; **, *p*<0.01 and ns, no significant difference. This experiment is representative of three individual experiments.


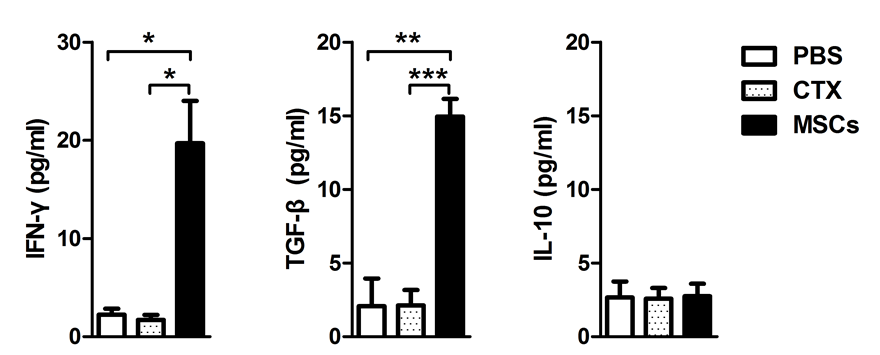


**Figure S4. MSC increased production of IFN-γ and TGF-β .** B6 mice were treated with MSCs or CTX and sacrificed at indicated time. PBS treated mice were used as controls. BALF were collected and the concentrations of IFN-γ, TGF-β and IL-10 were measured. Data were expressed as means ± SEM. n=4 mice for each treatment group; *, *p*<0.05; **, *p*<0.01; ***, *p*<0.001.


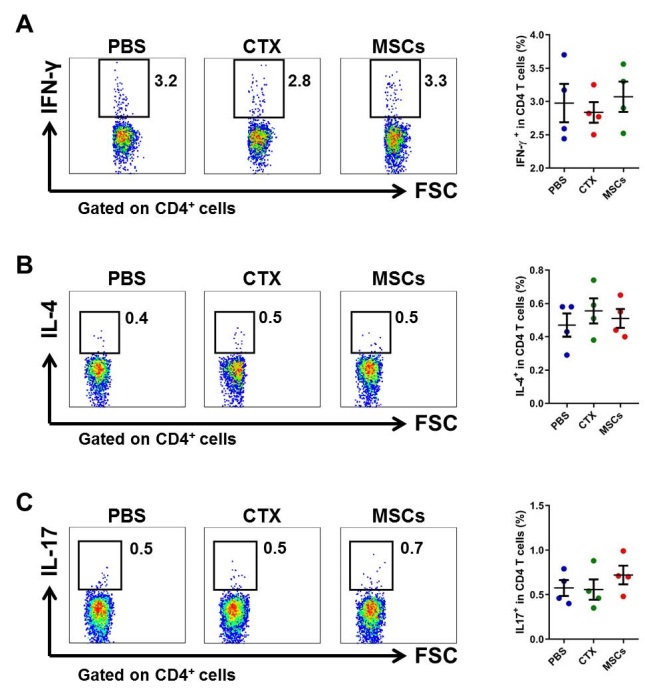


**Figure S5. Lung Th responses of infected mice.** Mice treated with MSCs or CTX were infected with Hi and sacrificed. Lung cells were isolated and stimulated with PMA and ionomysin for five hours. Cytokine production by CD4 T cells was determined by intracellular staining. Percentages of IFN-γ (A), IL-4 (B) and IL-17 (C) producing cells were shown. Data were expressed as means ± SEM. n=4 mice for each treatment group.
